# Neuroprotective properties of RT10, a fraction isolated from *Parawixia bistriata* spider venom, against excitotoxicity injury in neuron-glia cultures

**DOI:** 10.1590/1678-9199-JVATITD-1488-18

**Published:** 2019-05-16

**Authors:** Eduardo Octaviano Primini, José Luiz Liberato, Andreia Cristina Karklin Fontana, Wagner Ferreira dos Santos

**Affiliations:** 1 Neurobiology and Venoms Laboratory, Department of Biology, College of Philosophy, Sciences and Literature of Ribeirão Preto, University of São Paulo, Ribeirão Preto, SP, Brazil.; 2 Institute for Neurosciences and Behavior - INeC, Ribeirão Preto, SP, Brazil.; 3 Department of Pharmacology and Physiology, Drexel University College of Medicine, Philadelphia, PA, USA.

**Keywords:** *L-*Glutamate, excitotoxicity, neuroprotection, Parawixia bistriata, RT10

## Abstract

**Background:**

*L-*Glutamate (*L-*Glu), the major excitatory neurotransmitter in the mammalian Central Nervous System (CNS), is essential to cognitive functions. However, when *L-*Glu is accumulated in large concentrations at the synaptic cleft, it can induce excitotoxicity that results in secondary damage implicated in many neurological disorders. Current therapies for the treatment of neurological disorders are ineffective and have side effects associated with their use; therefore, there is a need to develop novel treatments. In this regard, previous studies have shown that neuroactive compounds obtained from the venom of the spider *Parawixia bistriata* have neuroprotective effects *in vitro* and *in vivo.* In this sense, this work aimed to evaluate potential neuroprotective effects of fraction RT10, obtained from this spider venom, on primary cultures of neuron and glial cells subjected to glutamate excitotoxicity insults.

**Methods:**

Primary cultures of neurons and glia were obtained from the cerebral tissue of 1-day-old postnatal Wistar rats. After 7 days *in vitro* (DIV), the cultures were incubated with fraction RT10 (0.002; 0.02; 0.2 and 2 µg/µL) or riluzole (100 µM) for 3-hours before application of 5 mM *L-*Glu. After 12 hours, the resazurin sodium salt (RSS) test was applied to measure metabolic activity and proliferation of living cells, whereas immunocytochemistry for MAP2 was performed to measure neuronal survival. In addition, the cells were immunolabeled with NeuN and GFAP in baseline conditions.

**Results:**

In the RSS tests, we observed that pre-incubation with RT10 before the excitotoxic insults from *L*-Glu resulted in neuroprotection, shown by a 10% reduction in the cell death level. RT10 was more effective than riluzole, which resulted in a cell-death reduction of 5%. Moreover, qualitative analysis of neuronal morphology (by MAP2 staining, expressed as fluorescence intensity (FI), an indirect measure of neuronal survival) indicate that RT10 reduced the toxic effects of *L*-Glu, as shown by a 38 % increase in MAP2 fluorescence when compared to *L*-Glu insult. On the other hand, the riluzole treatment resulted in 17% increase of MAP2 fluorescence; therefore, the neuroprotection from RT10 was more efficacious.

**Conclusion:**

RT10 fraction exhibits neuroprotective effects against *L*-Glu excitotoxicity in neuron-glia cultured *in vitro*.

## Background

*L-*Glutamate (*L-*Glu) is the major excitatory neurotransmitter in the Central Nervous System (CNS) of mammals [1]. Glutamatergic neurotransmission is fundamental to cognitive functions, such as learning and memory. Nevertheless, over-activation of *L-*Glu receptors leads to a hyperexcitability, which induces an excessive calcium (Ca2+) influx, which in turns results in excitotoxic-mediated cell death [2]. *L-*Glu transporters are responsible for glutamate clearance in the brain; therefore, they can be targeted for the development of therapies for CNS disorders, in which glutamate excitotoxicity plays an important role, such as Alzheimer’s disease, amyotrophic lateral sclerosis (ALS), autism, stroke, Huntington’s disease and epilepsy [2-5]. Another potential approach to development of therapies for these diseases would be an increase of GABAergic inhibitory neurotransmission, which may counteract the hyperexcitability of CNS mediated by *L-*Glu. In this context, compounds that either reduce *L-*Glu or enhance GABA concentrations at the synaptic cleft can provide therapeutic strategies against several neurological disorders. For instance, riluzole, a drug approved by the FDA (Food and Drug Administration) for ALS treatment, acts by inhibiting presynaptic release of *L-*Glu, preventing over-excitation of postsynaptic neurons and thus calcium influx, as well as intensifying GABAergic transmission, thereby exhibiting neuroprotective effects [6,7]. However, side effects associated with its use include anorexia, dizziness, diarrhea, headache and vomiting [8]. Thus, it is fundamental to seek new alternatives for treatments to provide a better life quality to the patients [9,10]. In this regard, venoms are natural sources for new compounds, since they present affinity and selectivity for a wide variety of targets in mammalian systems. Therefore, molecules derived from spider venoms that modulate CNS structures such as ion channels, receptors and transporters, should be studied and developed into therapies [11,12]. Furthermore, several pharmaceutical companies have developed venom-based drug discovery programs or used venom-derived molecules for target validation [13]. Our group has elucidated several biological and pharmacological activities of compounds isolated from the venom of the social spider *Parawixia bistriata (P. bistriata)* [14,15]. Some of these compounds display anxiolytic, anticonvulsant and neuroprotective effects, by acting on *L-*Glu and GABA (gamma-Aminobutyric acid) transporters [11,16,17]. In the present work, we investigated a potential neuroprotective effect of RT10, a pool of molecules from *P. bistriata* venom, in primary cultures of neuron and glial cells exposed to excitotoxic insults by the application of a high concentration of *L-*Glu.

## Methods

### Compound isolation

**Figure 1.**
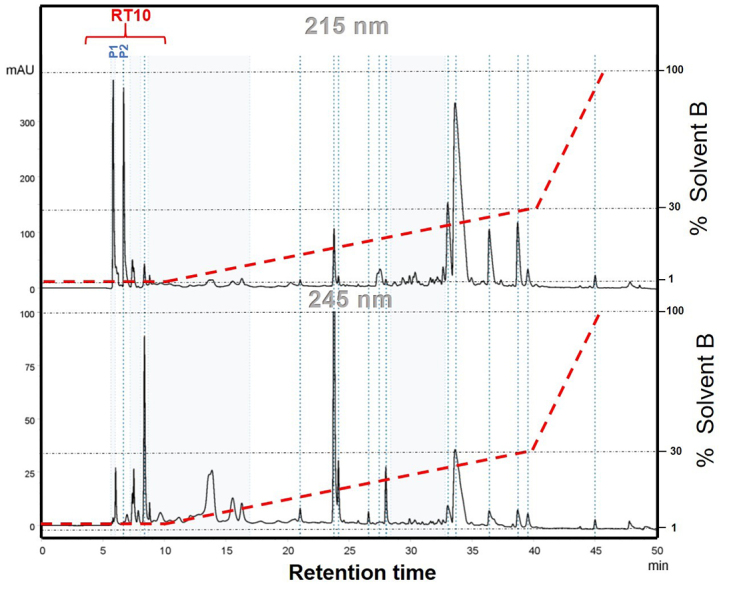
Representative chromatogram of the fractionation of *P. bistriata* spider venom in high-performance liquid chromatography (HPLC), with linear gradient of solvent B (Acetonitrile) from 1 to 100%. Flow rate in 8.0 ml/min and eluents monitored and recorded at 215 nm and 245 nm. RT10= fraction studied in this work. P1=Parawixin1; P2=Parawixin2.

Spiders were collected in the Brazilian state of São Paulo, near the city of Ribeirão Preto, according to Brazil’s Chico Mendes Institute for the Biodiversity Conservation (ICMBio- SISBIO protocol n° 46797). The specimens were euthanized by freezing at −20 °C. Venom glands and reservoirs were carefully removed and crushed in Milli-Q water, centrifuged at 3000×g for 10 min after which the supernatant was lyophilized and weighed. The resulting powder was submitted to fractionation by high performance liquid chromatography (HPLC; Shimadzu LC-6A; U.V. detector SPD-6AV; auto injector SI*L-*10ADvp, Shimadzu) using a previous methodology [18]. Briefly, lyophilized venom was dissolved in Milli-Q water (12 mg/mL) and applied onto a reverse-phase column (PREP-ODS 20×250 mm, 5 μm) previously equilibrated with 99% H_2_O (solvent A) and 1% (v/v) Acetonitrile (ACN, solvent B). This sample was eluted by solvent B in a linear gradient from 1 to 100%. The flow rate was 8.0 ml/min and elutes were continuously monitored at 215 and 245 nm. Eighteen fractions were collected and lyophilized, which included the fraction studied herein, referred to as RT10 (Figure 1).

### Cell culture 

We obtained a co-culture composed of neurons and astrocytes, since both cell types exhibit the potential targets for RT10 action, such as GABA and *L*-Glu transporters [19]. Therefore, the addition of Cytosine-β−D-arabinofuranoside (AraC), an anti-mitotic agent that intercalates DNA in the mitotic phase and halts non-neuronal cell proliferation, was omitted, allowing for glial growth. However, the exact proportion of glial and neuronal population in these cultures remains to be investigated. Primary cultures of neuron and glial cells were obtained from cortex and hippocampus, which are affected by glutamatergic excitotoxicity in several neurodegenerative diseases [5]; furthermore, we optimized the number of primary cells to be cultured, in order to reduce the number of animals in each experiment. Thus, the brain tissue from 1-day-old newborn Wistar rats was removed, dissociated and plated onto 24-well culture plates (coated with poly-L-lysine, with coverslips for immunocytochemistry analysis and without coverslips for viability tests), at the density of 5 x 10^5^cells per well, in Neurobasal-A medium (Gibco, USA) containing 2% B27 supplement (Gibco, USA), 1% antibiotic (Penicillin-Streptomycin, Invitrogen, USA) and 0.5 mM Glutamax supplement (Invitrogen, USA). Cultures were maintained at 37 °C, 5% CO_2_ in a humidified incubator until use with culture medium, which was changed every 2 to 3 days. Cells cultures were pretreated for 3 hours with either riluzole (100µM) or RT10 (at the following concentrations: 0.002; 0.02; 0.2 and 2 µg/µL) and then exposed to *L*-Glu (5 mM) for 12 hours in Locke’s solution at 7 DIV [20]. Subsequently, cell viability tests or immunocytochemistry was performed as described below.

### Viability assays

Cell viability in neuron-glia primary culture was assessed through the presence of 10% resazurin sodium salt (RSS, Sigma-Aldrich, USA), which is a non-toxic, permeable compound that is useful to measure metabolic activity and proliferation of living cells. RSS is a blue non-fluorescent dye that is reduced to the pink-colored, highly fluorescent resorufin, so that a higher fluorescence indicates a greater number of viable cells [21]. The fluorescence was measured in a spectrophotometer, with excitation at 530 nm and emission at 590 nm, 24 hours after *L-*Glu insults [22].

### Immunocytochemistry

Neurons and astrocytes were identified by immunocytochemistry labeling. We used the following primary antibodies: mouse anti-Neuronal Nuclei (NeuN; 1:500; Abcam, USA); rabbit anti-Microtubule associated protein 2 (MAP-2; 1:1000; Abcam, USA); chicken anti-Glial Fibrillary Acidic Protein (GFAP; 1:500; Abcam, USA) and secondary antibodies: goat anti-mouse IgG (Alexa Fluor 594; 1:1000, Abcam, USA); donkey anti-rabbit IgG (Alexa Fluor 488; 1:1000, Abcam, USA); rabbit anti-Chicken IgG (Alexa Fluor 405; 1:1000, Abcam, USA). The neurons and astrocytes immunolabeled with anti-NeuN and anti-GFAP were not exposed to *L*-Glu or treatments (RT10; riluzole). This part of immunostaining was performed to prove the presence of these cell types in the primary culture. However, we assessed the neuronal projections of cells affected by *L*-Glu exposure and treated by RT10 or riluzole, through anti-MAP2 immunolabeling. The images were analyzed by fluorescence microscopy (Leica Microsystems, Germany), with image acquisitions obtained via a camera system (Leica FX300; Leica Microsystems, Germany), coupled to the microscope with software for the acquisition and analysis of microscopic images, Leica Application Suite (LAS; Leica Microsystems, Germany).

### Data analysis

The software “Image J” (https://imagej.nih.gov/ij/; USA) was employed to obtain the fluorescence intensity (FI) of neurons labeled with anti-MAP2.In this study, the higher the fluorescence, the more neuronal projections were present in the cultures.

### Statistical analysis

The results from viability assays and FI for MAP2 immunocytochemistry were analyzed by ANOVA one way followed by Newman-Keuls test.

## Results

### High performance liquid chromatography (HPLC)

Fractionation of *P. bistriata* spider venom yielded, among other fractions, the RT10 fraction, which was characterized as a pool of compounds obtained in the first ten minutes of retention time and comprises Parawixin1 and Parawixin2 (Figure 1).

### Cellular viability

**Figure 2.**
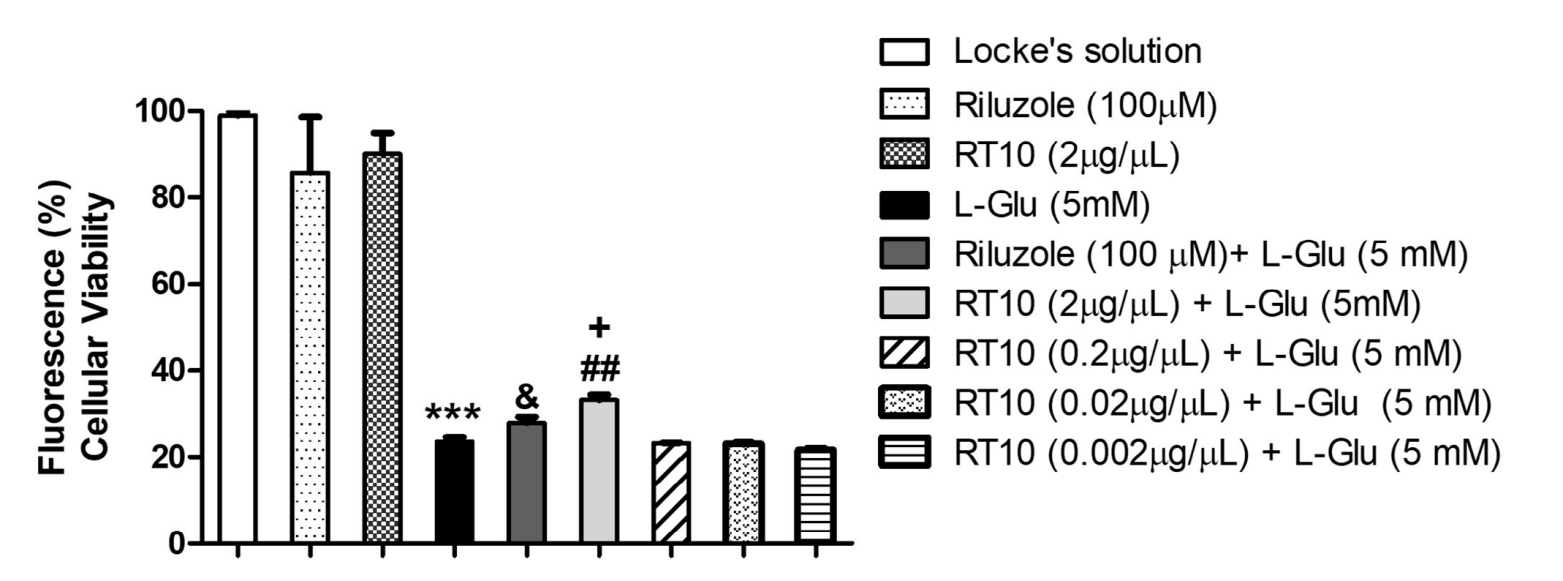
Cellular viability expressed in normalized levels of fluorescence (percent and standard deviation), after 12 hours incubation with *L-*Glu (5mM) to the cultures. Data is presented as mean ± S.E.M. Treatments with riluzole (100 μM) and RT10 (0.002; 0.02; 0.2 and 2 μg/μL) for 3h, before insults*,* produced insignificant differences. (***p< 0.001 vs. Locke’s solution group; &p<0.05 and ##p<0.01 vs. *L-*Glu-5 mM; +p<0.05 vs riluzole+*L-*Glu 5 mM). One-way ANOVA followed by Newman-Keuls test*.*

Quantifications by RSS tests (Figure 2) indicate that cell-death levels were significantly reduced (by 10%) by RT10 incubation at 2 μg/μL after the insult but were not altered by lower concentrations. Treatment with riluzole resulted in a neuroprotective effect, shown by a 5% reduction in the cell-death level when compared to insult alone. Cultures treated with riluzole and RT10 in the absence of insult did not alter the cell viability.

### Immunocytochemistry

**Figure 3.**
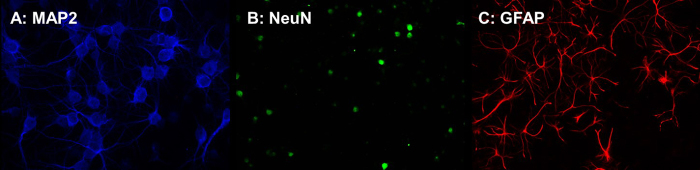
Immunofluorescence photomicrographs of primary cortical and hippocampus neuron/glia cultures in vitro (7 DIV) from Wistar rat brains. Anti-MAP2 (a) anti-NeuN (b) and anti-GFAP (c) in control conditions. 200x magnification.

**Figure 4.**
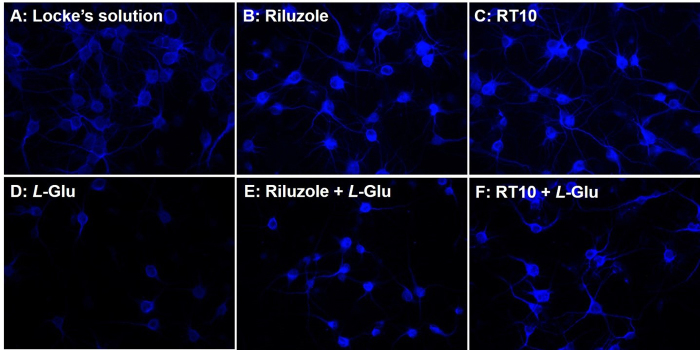
Immunological labeling with anti-MAP2 in primary cortical neuron/glia cultures. Treatments were performed three hours prior to incubation of *L*-Glu, which lasted for 12 hours. **A:** Locke's solution; **B:** Riluzole (100 μM); **C:** RT10 (2μg / ml); D) *L*-Glu (5 mM); E) Riluzole (100 μM) + *L*-Glu (5 mM); F) RT10 (2μg / ml) + L-Glu (5mM). 200x magnification.

**Figure 5.**
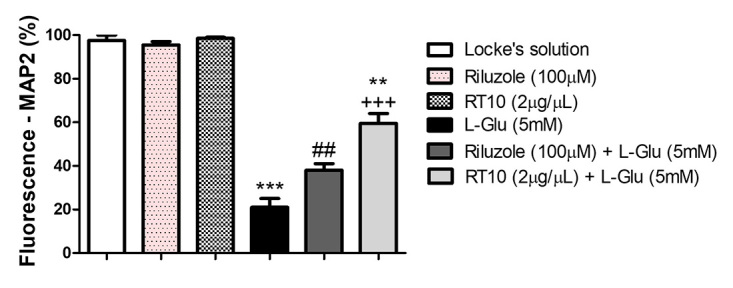
Normalized levels of FI in cells labeled with MAP2. Data are expressed as mean ± S.E.M. Cultures were treated with either riluzole (100 μM) or RT10 (2 μg/μL) for 3 hours prior to exposure to *L-*Glu (5mM) for 12 hours. (***p< 0.001 vs Locke’s solution group; ##p<0.01 vs. *L-*Glu; +++p<0.001 vs. *L-*Glu and **p<0.01 vs. riluzole). One-way ANOVA followed by Newman-Keuls test.

Neurons and astrocytes present in the primary culture under control conditions are displayed in Figure 3, labeled with anti-MAP2 (Figure 3A), anti-NeuN (Figure 3B) and anti-GFAP antibodies (Figure 3C). Representative images of cultures stained with MAP2 in different conditions are shown in Figure 4. Figure 4A (control conditions) shows the integrity of the neurons, whereas Figure 4B and Figure 4C show that incubation with riluzole and RT10, at the indicated concentrations, did not result in toxicity. In Figure 4D, incubation with *L-*Glu (5 mM) reduced the density of neurons (Figure 4D). On the other hand, incubation with riluzole (Figure 4E) and RT10 (Figure 4F) mitigated damage to cell projections, as shown by a higher density of anti-MAP2 labeling. Quantitative analysis (Figure 5) revealed that treatments with riluzole and RT10 were neuroprotective, as shown by respective increases of 17 and 38% in MAP2 fluorescence intensity (FI), in comparison with the insulted group. Treatments with riluzole and RT10 alone (Figure 4B, Figure 4C and Figure 5) did not result in significant differences between the FI and control cells.

## Discussion

Parawixins1 and 2 are included in fraction RT10 (Figure 1) [13,17,23]. RT10 was neuroprotective in the cultures subjected to excitotoxicity by 12 hours of exposure to *L*-Glu. Data displayed in figures 2, 4 and 5 suggest that RT10 protects neurons, as demonstrated by enhancing the MAP2 fluorescence, indicating less cellular death. It is important to highlight that MAP2 is found mostly in dendrites [24]. Accordingly, the results indicated that *L*-Glu caused a decline of these neuronal projections, which were preserved by RT10. We hypothesized that the observed neuroprotection resulted from previously reported activities of Parawixin1 and Parawixin2 in glutamate and GABA transporters, respectively [18,23]. Furthermore, Parawixin1 was shown to increase the function of Excitatory Amino Acid Transporter 2 (EAAT2), the main subtype of *L*-Glu transporter, which could explain the neuroprotective effects observed in the current work, due to the enhancement of transporter action that results in a lower amount of *L*-Glu in extracellular space, and consequent reductions of Ca^2+^influx and cell death [4-17]. Parawixin2 was shown to reduce GABA and Glycine uptake, but did not modulate the activity of Na^+^, K^+^ or Ca^2+^ions or of GABA receptors in synaptosomes from the cerebral cortex of rats [18]. Moreover, Parawixin2 demonstrated neuroprotection and anticonvulsant activities in animal models of temporal lobe epilepsy and acute glaucoma [13,18,25]. Since it is responsible for increasing of GABA in extracellular space, Parawixin2 potentiates the inhibitory neurotransmission in order to counterbalance the harmful overactivated glutamatergic neurotransmission [13,18,26]. We emphasized that several concentrations of RT10 were assessed in the assays of cellular viability in which the highest concentration (2 µg/µL) was most effective, consequently, this was evaluated in the immunocytochemistry assay for MAP2. In the present study, riluzole was used as the control treatment, given its neuroprotective effects and suitability for patients with ALS or other neuropathologies. This drug has been shown to act in several mechanisms against glutamatergic neurotransmission, such as enhancing the activity of *L*-Glu transporters in astrocytes, by inhibiting Ca^2+^influx in ion channels at the *L*-Glu receptors [10,27]. Furthermore, Mantz et al. [28] reported that riluzole presents inhibitory effects on GABA transporters. In the present study, RT10 revealed a greater neuroprotective effect than riluzole, as shown in quantitative and qualitative assays (Figure 2 and Figure 4). The mixed primary culture utilized in the current study was essential for investigating the neuroprotective effect of RT10, since Parawixin1 targets glutamatergic transporters and Parawixin2 targets GABA transporters. It is important to highlight the presence of transporters for glutamate and GABA in neurons and astrocytes, thereby highlighting the functional importance of the latter in the regulation of neurotransmitter levels in the synaptic cleft and hence for the activity of excitatory and inhibitory neurotransmission [29]. Immunocytochemistry and cellular viability assays were performed in 7 DIV cultures, a period in which mature neurons express the machinery that is susceptible to toxicity by *L*-Glu, for example the expression of the NR2A subunit of the NMDA (N-methyl-D-aspartate) receptor, which is essential for excitotoxicity and Ca2+ influx in a voltage-dependent channel [30]. In addition, Janssens and Lesage (2001) found that the NMDA receptor subunits (NR1, NR2A, and NR2B) were expressed in primary neuron cultures from rat cortex and hippocampus on the 7th DIV. We have not examined whether RT10 modulates the activity of NMDA receptors; such studies should be conducted in the future. The RT10 fraction, comprised of compounds Parawixin1 and Parawixin2, was studied under the premise supported by several studies [12,31,32] that drug action can involve plural targets to address disease in more subtle and effective ways. In the current work, we have demonstrated a neuroprotective effect of the RT10 fraction against excitotoxicity *in vitro.* Nevertheless, it remains to be evaluated whether a neuroprotective effect would be observed if RT10 were added after the insults. This study is an important step towards developing alternative treatments for neurological disorders, which are associated with glutamatergic excitotoxicity. Future investigations should elucidate other possible mechanisms of RT10 and ascertain possible neuroprotective properties in *in vivo* models.

## Conclusion

The RT10 fraction from *P. bistriata* spider venom is neuroprotective in neuroglia cultures exposed to toxic concentrations of *L*-Glu. In addition, the RT10 fraction exhibits a better effect than riluzole. These findings corroborate previous studies, in which Parawixin1 and Parawixin2, two constituents of RT10, were found to be neuroprotective and anticonvulsant in *in vivo* experimental models. Therefore, we consider RT10 to be a valuable tool for designing new drugs against neurodegenerative diseases.

### Abbreviations

ALS: Amyotrophic lateral sclerosis; FDA: Food and Drug Administration; FI: Fluorescence intensity; GABA: Gamma-Aminobutyric acid; GFAP: Glial Fibrillary Acidic Protein; HPLC: High performance liquid chromatography; *L-*Glu-*L*: Glutamate; MAP2: Microtubule associated protein 2; NeuN: Neuronal Nuclei; NMDA: N-methyl-D-aspartate; *P. bistriata*: *Parawixia bistriata*; P1: Parawixin1; P2: Parawixin2; RSS: Resazurin sodium salt.
